# 

**Published:** 1899-04

**Authors:** 


### INDEX TO VOLUME I.

---

ORIGINAL COMMUNICATIONS—

Abdomino-Perineal Procto-Sigmoidectomy .......... 380
Address of Welcome .......... 164
Appendicitis .......... 522
Appendicitis, An Interesting Case of .......... 452
Appendicitis, From Standpoint of Country Doctor .......... 289
Arsenical Paste, Painless Application of .......... 738
Blisters, Therapeutic Application of .......... 880
Breads Made with Different Leavening Agents, Relative Digestibility of .......... 793
Building of Atlanta, The .......... 649
Cancer of Skin, Treatment of Some Cases of .......... 248
Circumcision .......... 84
Climatology and Sanitation .......... 513
Conservative Gynecology, Some Cases in .......... 802
Deafness, Chronic, Something of the Tact and Patience Necessary in the Treatment of .......... 372
Diet in Lithemia .......... 734
Diphtheritic Croup, Seven Cases of .......... 241
Diseases of the Stomach, Diagnosis of .......... 884
Doughty, Memorial Address on the Late Dr. H. C .......... 159
Ear, Nose and Throat, General Consideration of Diseases of the .. 603
Electric Treatment, Different Phases of .......... 667
Electricity, Note on Treatment of Malignant Neoplasms by .......... 312
Electrolysis, Conditions Successfully Treated by .......... 662
Eye, Penetrating Wounds of the .......... 533
Fever, The Pathology of .......... 361, 433, 505
Fibroid Tumors of the Uterus .......... 73
Formaldehyde, A few Words in Reference to some Medical Uses of 93
Gonorrheal Patients, Advice to .......... 599
Heart-failure, Physiological Therapeutics of .......... 656
Law and Medicine .......... 889
Malpractice Suits, Protection against Fraudulent .......... 79
Meat and Milk, Dangers of Impure .......... 590
Medico-Religious Charity—The Guild of Mercy .......... 876
Membranous Obstruction of Larynx .......... 807
Mitral Stenosis .......... 445
Morphinism .......... 865
Multiple Neuritis from Arsenical Poisoning .......... 91
Nose and Throat Affections .......... 872
Pachymeningitis, A case of Chronic, Following Syphilitic Rhinitis 309

ORIGINAL COMMUNICATIONS—*continued*—

Pediatric Society, Necessity for State.... 305
Philippines, Some Notes on the.... 729
Reasoning Madness, Physiological Therapeutics of.... 721
Relation of the Board of Health to the City and the Medical Profession.... 651
Sarcomata of the Jaws, Some Cases of.... 577
Scarlet Fever and Diphtheria, How can Spread of, be Prevented in Public Schools.... 300
Speech of Hon. N. J. Hammond.... 145
Superstitions, Medical.... 456
Surgical Cases, Report of Three, with Comments.... 530
Suspension of the Uterus by Intramural Shortening of the Round Ligaments.... 537
Typhoid Fever, Report of Two Cases of.... 535
Typhoid Fever, Treatment of.... 217
Urethritis, Chronic, Endoscopic Treatment of.... 236
Whooping-cough, Treatment to Prevent Complications.... 811
X-ray in Surgery, Use and Abuse of the.... 87

SOCIETY REPORTS—

Atlanta Society of Medicine.... 95, 314, 384, 464, 547, 605, 816
Maryland, Clinical Society of.... 254, 676
Medical Association of Georgia.... 169
Memphis Medical Society, Proceedings of.... 104
Southern Surgical and Gynecological Association.... 740

CORRESPONDENCE—

American Medical Association—Annual Announcement.... 108
Confederate Veterans' Reunion at Louisville.... 899
Correspondents, Answers to.... 610
Letter from Dr. Ridley.... 824

EDITORIAL NOTES AND COMMENTS—

American Medical Association, Meeting of.... 333
Amo, I Love.... 481
Announcement.... 111
Antivivisection Movement, The.... 834
"As Ye Sow, That Shall Ye also Reap".... 265
Atlanta College of Physicians and Surgeons.... 112
Coddling of Children to Prevent Colds.... 114
Consumption, Isolation of.... 192
Germ Diseases and the Telephone.... 116
Gigantic Mistake, A.... 390
Homing Pigeons and the Country Doctor.... 903
Human Odor, The.... 835

EDITORIAL NOTES AND COMMENTS—*continued*—

"Husa" Sucker ........ 267
Liquid Air, Therapeutic Applications of ........ 480
Lombroso, Prof., and Vacher's Brain ........ 479
Medical Association of Georgia ........ 189
Medical Defense Unions ........ 619
Medical Examining Board ........ 194, 195
Medical News ........ 554
Metric System, The ........ 617
Mummified Meat ........ 268
Osteopathy Bill, Recent Veto of ........ 828
Osteopathy in Georgia, Rise and Fall of ........ 758
Parturition among the Esquimaux ........ 902
Philologic Reform ........ 616
Prolonged Standing, Evil Effects upon Women of ........ 613
Quackery, and a Suggestion ........ 900
Quackery in Georgia ........ 685, 831
Reading Character from the Inspection of the Vulva and Vagina. 388
Serum of Sobriety, The ........ 191
Southern Surgical and Gynecological Association ........ 761
State Board of Health ........ 688
Subscribers, To ........ 552
Witness, The Medical ........ 552
Words, Words, Words ........ 836

NEWS AND NOTES ........ 122, 201, 270, 339, 555, 621, 694, 766, 839, 907

MEDICAL MUSINGS ........ 132

LITERARY NOTES—

Gerrish's Anatomy, by American Authors ........ 206
May Ladies' Home Journal ........ 207

BOOK REVIEWS, PAMPHLETS, EXCHANGES—

*American Year-Book of Medicine and Surgery* (*Gould*) ........ 139
*American Text-Book of Surgery* (*Connor et al.*) ........ 773
*Anatomy, A Text-Book of* (*Gerrish*) ........ 341
*Annual and Analytical Cyclopedia of Practical Medicine* (*Sajous*). 774
*Appendicitis, Lectures on, and Notes on Other Subjects* (*Morris*) ... 140
*Atlas of External Diseases of the Eye* (*Haab*) ........ 342
*Bacteriology in Medicine and Surgery* (*Park*) ........ 848
*Chemistry: General, Medical and Pharmaceutical* (*Attfield*) ........ 275
*Compend of Practice of Medicine* (*Hughes*) ........ 849
*Cyclopedia of Practical Medicine* (*Sajous*) ........ 273
*Diagnosis of the Urine, or the Practical Examination of Urine with Special Reference to Diagnosis* (*Memminger*) ........ 913

BOOK REVIEWS, PAMPHLETS, EXCHANGES—*continued*—

*Diseases of the Eye* (*Schweinitz*) .... 143
*Diseases of the Eye, Ear, Nose and Throat, An American Text-Book of* (*Schweinitz and Randall*) .... 141
*Ear, Nose, Throat and Accessory Cavities, Diseases of* (*Bishop*) .... 273
*Experimental Research into Surgical Shocks, An* (*Crile*) .... 395
*Eye, Diseases of, and Refraction* (*Gould and Pyle*) .... 558
*Eye, Manual of Treatment of Diseases of* (*Jackson*) .... 847
*Fever-Nursing* (*Wilson*) .... 142
*Fractures and Dislocations, A Treatise on* (*Stimson*) .... 208
*Gynecology, Quiz Compend of* (*Wells*) .... 626
*Hay Fever and its Successful Treatment* (*Hollopeter*) .... 395
*History of Medicine, An Epitome of* (*Park*) .... 342
*Hyde on the Skin* .... 911
*Hygiene of Transmissible Diseases* (*Abbott*) .... 849
*International Medical Annual* .... 273
*Lea's Pocket Text-Book Series* .... 703
*Light of Reason, The* (*Franklin*) .... 143
*Magazine Notes* .... 775
*Medical Chemistry, Essentials of* (*Wolff*) .... 703
*Medical Complications, Accidents and Sequelæ of Typhoid Fever* (*Hare*) .... 394
*Mineral Waters of the U. S.* (*Crook*) .... 702
*Nervous and Mental Diseases* (*Church*) .... 208
*Nervous System, A Manual of Diseases of* (*Gowers*) .... 700
*Nervous System, Gross and Minute Anatomy of* (*Gordinier*) .... 702
*Newer Remedies, The* (*Coblentz*) .... 396
*Nose and Throat, A Text-Book of Diseases of* (*Kyle*) .... 483
*Nose and Throat, Diseases of* (*Coakley*) .... 557
*Obstetrics, A Text-Book on Practical* (*Grandin*) .... 209
*Obstetrics, The Practice of* (*Jewett*) .... 138
*Ophthalmology, A Text-Book of* (*Fuchs*) .... 393
*Pelvic Inflammation, Treatment of, through Vagina* (*Pryor*) .... 847
*Physical Diagnosis of the Thorax, The Essentials of* (*Cowan*) .... 911
*Physician's Visiting List for 1900* .... 704
*Physiological Chemistry* (*Rockwood*) .... 701
*Practice of Medicine, A Text-Book of* (*Anders*) .... 848
*Progressive Medicine* (*Hare*) .... 275
*Recollections of a Rebel Surgeon* (*Daniel*) .... 773
*Refraction and How to Refract* (*Thorington*) .... 702
*Refraction, the Nature and Consequences of Anomalies* (*Donders*). 210
*Sexual Impotence, Pathology and Treatment of* (*Vecki*) .... 701
*Skin, Atlas of Diseases of* (*Mraček*) .... 702
*System of Medicine, A* (*Albutt*) .... 625
*System of Medicine, A* (*by many writers*) .... 700
*The Surgical Diseases of the Genito-Urinary Tract, Venereal and Sexual Diseases* (*Lydston*) .... 912

BOOK REVIEWS, PAMPHLETS, EXCHANGES—*continued*—

*The Urine and Clinical Examination of the Gastric Contents, the Common Poisons and Milk* (*Holland*) .......... 913
*Treasure for Families, A* (*Price*) .......... 703

SELECTIONS AND ABSTRACTS—

Chlorosis, 211; Alcohol, Action on Respiratory Center of, 216; Tapeworm, Chloroform for, 216; Headache, Treatment of, 276; Laparotomy, Management of Patient Before and After, 280; Carcinoma of Rectum, Therapy of, 281; Cardiac Disease, Value of Rest in Treatment of, 282; War Investigation Committee, Report of, 284; Hay Fever, Prevention of, 285; Medical Cabinet Officer, A, 287; Carbolic Acid, Alcohol an Antidote for, 287; Menstruating Man, A, 288; Mosquitoes and Malaria, 343; Dont's for Treatment of Pneumonia, 347; Time Element in Therapeutics, The, 349; Leucemia, Splenic, Retinal Hemorrhage in, 350; Scarlatina, Prophylactic Gargle in, 350; Journalism, Medical, 350; Sleep in Treating Disease, 351; Urethritis, Posterior, Treatment of, 353; Boric Acid and Borax, Dermatitis from, 355; Prescriptions, 356, 357; Asthma, Treatment of, 358; European Medicine About 1799, 397; Patella, Treatment of, Fractures of the, 413; Prominent Persons, The Causes of Death of, 421; Syphilis—How Shall we Stop its Alarming Spread? 425; Time Limit, 428; Standardization Needed, 431; Cancer, Pathology and Therapy of, 484; Typhoid Fever Among our Soldiers in the Late War, 500; Michigan Medical Laws, 501; Christian Science, The Phallic Phase of, 503; Uterine Fibroids, Treatment of, 504; Shock, How to Treat, 504; Abdominal Contusions and Hollow Visceral Lesions, Diagnosis of, 559; La Grippe, 566; Surgical Hints, 568; Sodium Bromide Cure of Morphine, 569; Absence of Restraining Power in Man, 570; Hydrocele of Tunic, New Operation for, 571; Urea, 570; Tetanus, Successfully Treated, 571; Schleich's General Anesthesia, 572; Chancre of Syphilis, The, 573; Constipation, 573; Typhoid Germs in the Atmosphere, 574; Peritonitis, General Septic, 574; Hot Water Drinking, 575; Eye, The Itching, 575; Sneezing, Paroxysmal, 576; Giving Quinine, 576; Enema, An Elephantine, 576; Frictions with Hot Salt, 576; Hysteria and Organic Disease of Nervous System, 626; Stomach Tube, 626; Puerperal Insanity, 634; Liver and Biliary Tract, Wounds of, 637; Constipation, The Treatment of, 639; Uterus, Rupture of, Causes, 643; Mark Twain on Christian Science, 644; Twelfth Census of U. S., Vital Statistics, 646; House to House Operating, 647; Beriberi in the United States, 648; Success in the Practice of Medicine, 704; Cancer, Increase of, 706; Bone and Joint Disease, Certain Aspects of, 709; Constipation, Obstinate, Significance of, 712; Cancer, Cutaneous Arsenical Treatment of, 714; Character of the Author of "Science and Health," 713; Life Insurance Examiner, 715; Craig Colony Prize, 716; Conservation in Pelvic Surgery, 717; Medical Defence Union, 718; Tympanic Membrane, New Method of Anesthesia of, 719; Death in Consequence of Prolonged

SELECTIONS AND ABSTRACTS—*continued*—

Laughter, 719; Death-Dealing Long Tube Nursing Bottle, 720; Opium Habit, Cure for, 720; Iritis, Syphilitic, 720; Life Insurance, Medical Aspect of, 775; Pretuberculous Stage, Diagnosis of, 784; Medical Pedagogics, 786; Osteopaths Must Leave Kentucky, 788; Bladder, Accidental Wounds of Female, 789; Chicken Broth, 792; Morphine before Anesthetics, 792; Genital Tract in Women, Nature and Treatment of Acute Infections of, 849; Typhoid Fever, Mortality of in Principal Cities of United States, 855; The Wounded in South Africa, 857; Abdominal Exploration, 860; Progress in Therapeutics, 863; Retroversion of the Uterus, The Reflex Symptoms of, 913; Puerperal Fever, Treatment of, 918; Microcephalic Idiocy, Craniectomy for, 923; Suprarenal Extract in Rhinology, Notes on the Use of, 925; Heart Disease, Digitalis in the Treatment of, 928; Self-Medication, 929; Tonsillitis and Rheumatism, 930; Epilepsy, The Relation of Migraine to, 931; Pain-enduring Animals, 932; An Interesting Experiment, 933; Serotherapy and Organotherapy among Ancient Greeks, 934; Digitalis Poisoning, The Treatment of, 935; Ants in Surgery, 936; Baths for Infants, 936.

### LIST OF CONTRIBUTORS TO VOLUME I.

| | |
|---|---|
| Hardon, V. O. | Atlanta, Ga. |
| Wolff, Bernard | Atlanta, Ga. |
| Hancock, Thos. H | Atlanta, Ga. |
| Gaston, J. McF. | Atlanta, Ga. |
| Gaston, J. McF., Jr | Atlanta, Ga. |
| Harrison, Isham | Deerbrook, Miss. |
| Hudson, W. H | Lafayette, Ala. |
| Hammond, N. J | Atlanta, Ga. |
| Allen, Jos. Eve | Augusta, Ga. |
| Speer, Emory | Macon, Ga. |
| Hare, H. A | Philadelphia, Pa. |
| Champion, W. L | Atlanta, Ga. |
| Harbin, R. M | Rome, Ga. |
| Hutchins, M. B | Atlanta, Ga. |
| Davidson, A. C | Sharon, Ga. |
| Robinson, Gilman | Atlanta, Ga. |
| Bush, Oliver B | Colquitt, Ga. |
| Corson, Eugene R | Savannah, Ga. |
| Johnson, J. Clarence | Atlanta, Ga. |
| Hobbs, Arthur G | Atlanta, Ga. |
| Carson, Marcus F | Griffin, Ga. |
| Ellis, James N | Atlanta, Ga. |
| Moncrief, W. H | Atlanta, Ga. |
| Conway, D. W. B. | Athens, Ga. |
| Hopkins, T. S. | Thomasville, Ga. |
| Lorimer, H. S | Fair Haven, Ohio. |
| Davis, E. C | Atlanta, Ga. |
| Patterson, A. B | Atlanta, Ga. |
| McClanahan, J. M | Retreat, S. C. |
| Noble, Geo. H | Atlanta, Ga. |
| Dalrymple, W. H | Baton Rouge, La. |
| Cox, W. J | Gainesville, Ga. |
| Kime, R. R | Atlanta, Ga. |
| Lindorme, C. A. F | Chicago, Ill. |
| Bourns, F. S | Atlanta, Ga. |
| Conklin, A. B | Springfield, O. |
| Nicolson, W. P | Atlanta, Ga. |
| Everhart, Edgar | Atlanta, Ga. |
| Massey, G. Betton | Philadelphia, Pa. |
| Bell, W. Jay | Atlanta, Ga. |
| Murphey, C. E | Atlanta, Ga. |

Stockard, C. C. . . . . . . . . . . Atlanta, Ga.
Powell, C. H. . . . . . . . . . . St. Louis, Mo.
Parker, W. Thornton. . . . . . . . . . . Westboro, Mass.
Hurt, C. D. . . . . . . . . . . Atlanta, Ga.
Aaron, Charles D. . . . . . . . . . . Detroit, Mich.

***NOTICE.—This JOURNAL and THE INTERNATIONAL JOURNAL OF SURGERY one year for $1.00 cash, to new subscribers. No out-of-town checks accepted unless 10c. is added for cost of cashing.***

### CORRESPONDENCE.

### AMERICAN MEDICAL ASSOCIATION—ANNUAL ANNOUNCEMENT.

Office of the Permanent Secretary,
1400 Pine St., Philadelphia.

The fiftieth* annual session will be held in Columbus, Ohio, on Tuesday, Wednesday, Thursday and Friday, June 6, 7, 8 and 9, commencing on Tuesday, at 11 A.M.

"The delegates shall receive their appointment from permanently organized State medical societies, and such county and district medical societies *as are recognized by representation in their respective State Societies*, and from the Medical Departments of the Army, Navy, and Marine-Hospital Service of the United States.

"Each State, county and district medical society entitled to representation shall have the privilege of sending to the Association one delegate for every ten of its regular resident members, and one for every additional fraction of more than half that number: *provided*, however, that the number of delegates for any particular State, Territory, county, city or town shall not exceed the ratio of one in ten of the resident physicians who may have signed the Code of Ethics of the Association."

*Members by Application.*—Members by application shall consist of such members of the State, county and district medical societies entitled to representation in this Association, as shall make application in writing to the Treasurer, and accompany said application with a certificate of good standing, signed by the president and secretary of the society of which they are members, and the amount of the annual membership fee, $5. They shall have their names upon the roll, and have all the rights and privileges accorded to *permanent members*, and shall retain their membership upon the same terms.

* No meetings in 1861 and 1862.

At a recent meeting of this Association the following was unanimously adopted:

WHEREAS, The American Medical Association did, at Detroit, in 1892, unanimously resolve to demand of all the medical colleges of the United States the adoption and observance of a standard of requirements of all candidates for the degree of doctor of medicine which should in no manner fall below the minimum standard of the Association of American medical colleges; and

WHEREAS, This demand was sent officially by the permanent Secretary to the deans of every medical college in the United States and to every medical journal in the United States, now therefore the American Medical Association gives notice that hereafter no professor or other teacher in, nor any graduate of, any medical college in the United States, which shall after January 1, 1899, confer the degree of doctor of medicine or receive such degree on any conditions below the published standard of the Association of American medical colleges, will be allowed to register as either delegate or permanent member of this Association.

Each delegate or permanent member, when he registers, is requested to record the name of the Section, if any, that he will attend, and in which he will cast his vote for section officers.

Secretaries of medical societies, as above designated, are earnestly requested to forward, *at once*, lists of their delegates.

Also, that the permanent Secretary may be enabled to erase from the roll the names of those who have forfeited their membership, the Secretaries are, *by special resolution*, requested to send to him, annually, a corrected list of the membership of their respective societies.

### ORATIONS.

On Medicine, James C. Wilson, Philadelphia; on Surgery, Floyd W. McRae, Atlanta, Ga.; on State Medicine, Daniel R. Brower, Chicago; Chairman Committee of Arrangements, Starling Loving, Columbus.

### AMENDMENT.

Offered by W. L. Wills, Cal.:

Constitution, Art. IV.—Officers. Amend to read: "The following officers, viz.: President, four Vice-Presidents, Treasurer, Librarian, Secretary, Assistant Secretary, and Chairman of Committee of Arrangements, shall be nominated by a special committee of one member from each State represented at the meeting, and shall be elected annually by the vote on a joint ticket, and shall hold office until their successors are elected."

SECTIONS.

"The chairman of each section shall prepare an address on the recent advancements in the branches belonging to his section, including such suggestions in regard to improvements or methods of work as he may regard important, and present the same, on the first day of the annual meeting, to the section over which he presides. The reading of such address not to occupy more than forty minutes."—*By-Laws.*

"A member desiring to read a paper before a section should forward the paper, or its title and length (not to exceed twenty minutes in reading), to the secretary of the section, at least one month before the annual meeting at which the paper or report is to be read."—*By-Laws.*

OFFICERS OF SECTIONS.

*Practice of Medicine.*—Frank Billings, Chicago, Chairman; Carroll E. Edson, Denver, Secretary.

*Surgery and Anatomy.*—W. J. Mayo, Rochester, Minn., Chairman; M. L. Harris, Chicago, Secretary.

*Obstetrics and Diseases of Women.*—A. H. Cordier, Kansas City, Mo., Chairman; W. D. Haggard, Jr., Nashville, Tenn., Secretary.

*Materia Medica, Pharmacy and Therapeutics.*—Thomas H. Stucky, Louisville, Ky., Chairman; Leon L. Solomon, Louisville, Ky., Secretary.

*Ophthalmology.*—Casey A. Wood, Chicago, Chairman; Charles H. Williams, Boston, Secretary.

*Laryngology and Otology.*—Emil Mayer, New York, Chairman; Christian R. Holmes, Cincinnati, Secretary.

*Diseases of Children.*—Henry E. Tuley, Louisville, Ky., Chairman; L. D. Boogher, St. Louis, Secretary.

*Physiology and Dietetics.*—J. Weir, Jr., Owensboro, Ky., Chairman; Lee Kahn, Leadville, Colo., Secretary.

*Neurology and Medical Jurisprudence.*—Frederick Peterson, New York, Chairman; Hugh T. Patrick, Chicago, Secretary.

*Cutaneous Medicine and Surgery.*—W. T. Corlett, Cleveland, Ohio, Chairman; J. M. Blaine, Denver, Colo., Secretary.

*State Medicine.*—Arthur R. Reynolds, Chicago, Chairman; W. P. Munn, Denver, Colo., Secretary.

*Stomatology.*—George V. I. Brown, Milwaukee, Wis., Chairman; Eugene S. Talbot, Chicago, Secretary.

WILLIAM B. ATKINSON, Permanent Secretary.

### REPRINTS RECEIVED.

The Serum Treatment of Diphtheria. By William Cheatham, M.D., Louisville.

The Eighth Annual Report of the New Orleans Eye and Ear Infirmary.

The Use of Gloves in Surgery. By W. R. Lockett. From the Laboratories of the Jefferson Medical College Hospital.

Holocain in Ophthalmic Surgery. By Haskett Derly, M.D., Boston.

The Light of Reason. A Solution of the Economic Riddle. By A. B. Franklin.

Notes on the Absorption versus the Digestion of Milk. By L. Duncan Bulkley, A.M., M.D., N. Y.

The Etiology and Diagnosis of Empyema of the Accessory Sinuses of the Nose. By Jacob E. Schadle, M.D., St. Paul, Minn.

Prostatectomy. By Parker Syms, M.D., New York.

Twenty-ninth Report of the Alexian Brothers' Hospital. St. Louis.

Perichondritis and Necrosis of the Arytenoid Cartilage. By W. Scheppegrell, New Orleans.

The Galvanic Current for the Treatment of Pterygium. By Horace M. Starkey, M.D., Chicago.

Thirteenth Report of the Lunacy Commission of the State of Maryland.

Michigan Monthly Bulletin of Vital Statistics. January.

The Twelfth Annual Announcement of the New Orleans Polyclinic.

Pelvic Suppuration. By Byron B. Davis, of Omaha, Neb.

Malignant Disease of the Kidney. By Byron B. Davis, Omaha, Neb.

An Ephemeris of Materia Medica, Pharmacy, Therapeutics and Collateral Information. By E. R. Squibb, M.D.; E. H. Squibb, M.D.; C. F. Squibb, A.B., Brooklyn.

Twenty-first Annual Report of the Presbyterian Eye, Ear and Throat Hospital, of Baltimore, Md.

---

***NOTICE.—This JOURNAL and THE INTERNATIONAL JOURNAL OF SURGERY one year for $1.00 cash, to new subscribers. No out-of-town checks accepted unless 10c. is added for cost of cashing.***

